# Comparative Safety Profiles of Sacituzumab Govitecan and Datopotamab Deruxtecan: A Real-World Pharmacovigilance Study

**DOI:** 10.3390/jcm15197405

**Published:** 2026-09-23

**Authors:** Ziya Kalkan, Murat Bardakci, Tugba Tezer Kalkan, Yakup Ergun

**Affiliations:** 1 Department of Medical Oncology, Faculty of Medicine, Dicle University, Diyarbakır 21280, Türkiye; 2 Department of Medical Oncology, Gazi Yaşargil Training and Research Hospital, Diyarbakır 21010, Türkiye; 3 Department of Medical Oncology, Bower Hospital, Diyarbakır 21100, Türkiye

**Keywords:** antibody–drug conjugate, TROP2, sacituzumab govitecan, datopotamab deruxtecan, pharmacovigilance, AEMS

## Abstract

**Background:** Sacituzumab govitecan (SG) and datopotamab deruxtecan (Dato-DXd) are TROP2-directed antibody–drug conjugates with topoisomerase I inhibitor payloads but distinct recognized adverse-event profiles. Comparative reporting patterns for these agents remain incompletely characterized. **Methods:** We analyzed a case-level line listing downloaded from the U.S. Food and Drug Administration (FDA) Adverse Event Monitoring System (AEMS; formerly the FDA Adverse Event Reporting System [FAERS]) Public Dashboard on 7 July 2026. Reports listing SG or Dato-DXd among suspect product names or active ingredients were assigned mutually exclusively. Prespecified toxicity phenotypes and individual MedDRA Preferred Terms (PTs) were compared using reporting proportions, comparative reporting odds ratios (RORs), false-discovery-rate correction, and multivariable logistic regression. A breast cancer-restricted analysis with crude RORs and 95% CIs was also performed. **Results:** The cohort comprised 6368 distinct Case IDs (SG, 5714; Dato-DXd, 654). Compared with SG, Dato-DXd reports were enriched for mucositis/stomatitis (18.3% vs. 1.3%; ROR, 17.61; adjusted reporting odds ratio [aROR], 20.04) and ocular toxicity (10.9% vs. 0.9%; ROR, 13.26; aROR, 13.98). The aRORs for Dato-DXd versus SG were 0.11 for hematologic cytopenia, 0.12 for neutropenia/febrile neutropenia, 0.11 for diarrhea/colitis, and 0.57 for infection/sepsis. Exact interstitial lung disease/pneumonitis showed reporting enrichment with Dato-DXd in the unadjusted analysis (ROR, 3.12), but the comparative reporting association was attenuated after adjustment (aROR, 1.44; 95% CI, 0.87–2.39). Overall gastrointestinal reporting was similar, and the breast cancer-restricted crude RORs were directionally consistent with the full-cohort comparisons. **Conclusions:** SG and Dato-DXd showed distinct adverse-event reporting profiles. SG reports were characterized by relative enrichment of hematologic and diarrheal events, whereas Dato-DXd reports were enriched for mucosal and ocular events. These findings are hypothesis-generating reporting comparisons and should not be interpreted as incidence estimates, causal associations, or conventional head-to-head safety estimates.

## 1. Introduction

Antibody–drug conjugates (ADCs) integrate the target selectivity of monoclonal antibodies with the cytotoxic activity of potent payloads and have become an established therapeutic platform in solid tumors [1]. Trophoblast cell-surface antigen 2 (TROP2), a transmembrane glycoprotein expressed in several epithelial malignancies, is a clinically validated ADC target, particularly in breast cancer [2]. Safety, however, is determined by the complete ADC construct rather than by target expression alone. Antibody structure, linker stability, payload characteristics, drug-to-antibody ratio, pharmacokinetics, and systemic payload exposure may all influence the distribution and severity of adverse events [3]. Sacituzumab govitecan (SG) couples a humanized anti-TROP2 antibody to SN-38 through a hydrolyzable linker, whereas datopotamab deruxtecan (Dato-DXd) uses an exatecan-derived payload attached through a plasma-stable, tumor-selective cleavable linker [4,5].

Randomized trials have established the antitumor activity of both agents while identifying clinically distinct adverse-event profiles. In ASCENT-03, SG improved progression-free survival compared with chemotherapy in previously untreated advanced triple-negative breast cancer among patients who were not candidates for immune checkpoint inhibition; neutropenia and diarrhea were among the principal toxicities [6]. In TROPION-Breast02, Dato-DXd improved progression-free and overall survival compared with investigator’s-choice chemotherapy in a related first-line setting, while stomatitis, ocular adverse events, and interstitial lung disease (ILD) or pneumonitis required specific preventive and monitoring strategies [7]. As TROP2-directed ADCs enter broader indications and earlier treatment lines, a clearer characterization of their relative reporting profiles may help define surveillance priorities without substituting for comparative trial evidence.

The US Food and Drug Administration (FDA) adverse-event reporting infrastructure, now accessible through the FDA Adverse Event Monitoring System (AEMS; formerly the FDA Adverse Event Reporting System [FAERS]) Public Dashboard, provides report-level pharmacovigilance data from heterogeneous clinical settings [8,9]. Disproportionality analyses of such data can identify reporting patterns and generate safety hypotheses, but they cannot determine incidence, absolute risk, or causality because exposed population denominators are unavailable and reporting is affected by underreporting, stimulated reporting, missing information, duplication, and confounding [10,11]. These constraints are particularly relevant when drugs with different launch dates, indications, and regulatory histories are compared.

Previous pharmacovigilance studies have described hematologic and gastrointestinal reporting signals with SG, whereas clinical experience with Dato-DXd has emphasized mucosal, ocular, and pulmonary adverse events [12,13]. Direct report-level comparisons between the two TROP2-directed ADCs remain limited. We therefore compared their adverse-event reporting profiles using prespecified toxicity phenotypes, PT-level screening, false-discovery-rate correction, multivariable adjustment, and a breast cancer-restricted analysis. The objective was to characterize relative reporting enrichment and to inform clinical vigilance, not to rank the agents by overall safety.

## 2. Materials and Methods

### 2.1. Study Design and Data Source

We conducted a retrospective, report-level pharmacovigilance study using a line-listing file downloaded from the FDA AEMS Public Dashboard on 7 July 2026 [9]. This date was treated as the data-lock date. The findings therefore reflect the dashboard content available at extraction and exclude reports entered or modified subsequently.

The line listing contained Case ID, suspect product names, suspect product active ingredients, reason for use, MedDRA PT-coded reactions, seriousness, outcomes, sex, age, event date, FDA received dates, reporter type, report source, country of event, concomitant products, manufacturer fields, and literature references. The export identified products listed as suspect but did not distinguish primary from secondary suspect roles. No restriction by report source was applied. Accordingly, the analytic cohort should not be interpreted as consisting exclusively of spontaneous primary-suspect reports. The file was analyzed as provided and was not linked to prescription, sales, claims, electronic health record, trial, or other exposure-denominator data.

### 2.2. Cohort Definition

Reports were eligible when the suspect product name or active ingredient field contained a prespecified identifier for either ADC. SG was identified using sacituzumab govitecan, sacituzumab govitecan-hziy, or Trodelvy; Dato-DXd was identified using datopotamab deruxtecan, datopotamab deruxtecan-dlnk, or Datroway. Reports were assigned mutually exclusively to one comparator. Records containing both ADCs were prespecified for exclusion; none were identified.

Duplicate review was performed at the Case ID level. All Case IDs retained in the analytic file were distinct. Because separate Case IDs may nevertheless represent parallel or duplicate submissions of the same clinical event, Case ID uniqueness was not equated with patient-level uniqueness. SG was the reference group and Dato-DXd the comparator throughout the disproportionality and regression analyses.

### 2.3. Report-Level Variables

Age, sex, reporter type, event country, report year, indication, seriousness, and outcome were extracted from the line listing. Age was converted to years according to the reported unit, including years, decades, months, weeks, and days. Values recorded as Not Specified were treated as missing. Age was modeled per 10-year increase and centered at the median observed age of 57 years. For reports with missing age, the centered age term was set to zero and a separate missing-age indicator was included. This approach retained reports with unavailable age in the regression models; no multiple or model-based imputation of the unobserved age values was performed. Sex was categorized as female, male, or not specified. Reporter type was dichotomized as healthcare professional versus non-healthcare professional or other. Event country was summarized descriptively and coded as United States versus non-United States or unspecified for adjusted modeling. Report year was derived from the latest FDA received date.

Indication was derived from the Reason for Use field. Breast cancer reports were identified using breast cancer-related terms, including metastatic breast cancer, triple-negative breast cancer, hormone receptor-positive/HER2-negative breast cancer, HER2-positive breast cancer, and other breast carcinoma descriptors. Lung cancer reports were identified using lung cancer, non-small cell lung cancer, metastatic non-small cell lung cancer, and related terms. Seriousness was taken directly from the Serious field. Reports containing Died in the Outcomes field were classified as Outcome = Died.

### 2.4. Adverse-Event Phenotype Mapping

The Reactions field was parsed into individual MedDRA PTs using semicolons as delimiters. A report was counted once within a toxicity phenotype when at least one constituent PT was present; multiple PTs within the same phenotype did not increase the report-level count.

The phenotype-mapping framework considered the following candidate domains: broad pulmonary toxicity; exact ILD/pneumonitis; hematologic cytopenia; neutropenia/febrile neutropenia; standalone febrile neutropenia; infection/sepsis; overall gastrointestinal toxicity; diarrhea/colitis; nausea/vomiting; mucositis/stomatitis; ocular, hepatic, cardiac, neurologic, renal, and skin toxicity; fatigue/asthenia; and disease progression/drug ineffective-coded reports.

The primary comparative phenotype-level analyses were restricted to eight clinically prioritized and interpretable phenotypes: mucositis/stomatitis, ocular toxicity, exact ILD/pneumonitis, hematologic cytopenia, neutropenia/febrile neutropenia, diarrhea/colitis, infection/sepsis, and overall gastrointestinal toxicity. Broad pulmonary toxicity, standalone febrile neutropenia, and nausea/vomiting were not reported as separate composite outcomes because they overlapped with the selected pulmonary, hematologic, or gastrointestinal phenotypes. Standalone febrile neutro-penia was additionally evaluated at the individual PT level (Supplementary Table S2). Hepatic, cardiac, neurologic, renal, skin, and fatigue/asthenia categories were retained as exploratory mapping domains but were not carried forward into the primary composite-level comparison because of their heterogeneous PT composition and the focused comparative scope of the study. Disease progression/drug ineffective-coded reports were retained in PT-level screening but were not interpreted as toxicity outcomes.

Selection of the eight primary phenotypes was based on clinical relevance, phenotype specificity, and avoidance of overlapping outcomes rather than on the statistical significance of the observed results. All individual PTs meeting the prespecified ≥10-report threshold, including eligible PTs from the non-primary domains, are reported in Supplementary Table S2. Death as a reaction PT was evaluated separately as a descriptive reporting variable.

The exact ILD/pneumonitis phenotype comprised the PTs Interstitial Lung Disease and Pneumonitis. Mucositis/stomatitis included oral and mucosal inflammatory PTs. Ocular toxicity included dry eye, keratitis, blurred vision, visual impairment, and related eye PTs. Hematologic cytopenia included neutropenia, febrile neutropenia, anemia, thrombocytopenia, leukopenia, pancytopenia, myelosuppression, and relevant count-decrease PTs. Diarrhea/colitis included diarrhea, colitis, enterocolitis, and related intestinal inflammatory PTs. Complete PT definitions for the eight selected toxicity phenotypes are provided in Supplementary Table S1. Administrative or operational PTs, including Off Label Use, No Adverse Event, Inappropriate Schedule of Product Administration, and Prescribed Underdose, were retained in PT-level screening but were distinguished from clinical adverse-event PTs.

### 2.5. Statistical Analysis

All statistical analyses were performed using Python (version 3.13; Python Software Foundation, Wilmington, DE, USA). Categorical variables are reported as counts and percentages and continuous variables as medians with interquartile ranges (IQRs). For each phenotype, the reporting proportion was the number of reports containing that phenotype divided by the total number of reports assigned to the same ADC.

The primary comparative measure was the comparative reporting odds ratio (ROR) for Dato-DXd versus SG. For each phenotype or PT, a 2 × 2 table was constructed, and the ROR was calculated as (a/b)/(c/d), where a and b represented Dato-DXd reports with and without the event and c and d represented SG reports with and without the event, respectively; SG was the reference group. Accordingly, ROR in this study denotes a direct comparison between the two drug cohorts rather than the conventional comparison of a drug–event pair with the remainder of the AEMS database. RORs are presented with 95% confidence intervals (CIs). Fisher’s exact test was used when any expected cell count was <5; Pearson’s chi-square test was used otherwise. *p* values were adjusted using the Benjamini–Hochberg false-discovery-rate procedure within each prespecified analysis family.

Multivariable logistic regression models were fitted for the eight selected phenotypes, with phenotype presence as the dependent variable and ADC type as the primary independent variable. Each model included the same full cohort of 6368 reports (SG, *n* = 5714; Dato-DXd, *n* = 654) and was adjusted for age per 10-year increase, a missing-age indicator, sex, report year, breast cancer indication, lung cancer indication, healthcare professional reporter status, and United States event country. Reports with unavailable age were retained using the missing-age-indicator approach described above; therefore, the effective sample size was identical across all eight models. Estimates for Dato-DXd relative to SG are reported as adjusted reporting odds ratios (aRORs) with 95% CIs, nominal *p* values, and FDR-adjusted q values. Report year was modeled continuously per one-year increase to account for secular calendar-time trends in reporting.

PT-level analysis was exploratory and restricted to PTs reported in at least 10 records in the full cohort. All PTs meeting this threshold constituted a single PT-level family for Benjamini–Hochberg FDR correction, and the complete results are provided in Supplementary Table S2. The main clinically interpretable PT table emphasizes representative clinical terms relevant to the prespecified toxicity domains; administrative or operational PTs were screened but not presented as clinical toxicity signals.

### 2.6. Sensitivity and Descriptive Analyses

As a sensitivity analysis addressing differences in reporting maturity, the same multivariable models and missing-age approach were repeated after restricting the cohort to the common reporting period of 2025–2026 (Supplementary Table S3). Within this subset, crude comparative RORs with 95% CIs were calculated for the eight selected toxicity phenotypes using the same 2 × 2 contingency-table approach and statistical test-selection rule as in the primary analysis. *p* values were adjusted across the eight subgroup phenotype comparisons using the Benjamini–Hochberg procedure. Outcome = Died and Death as a reaction PT were analyzed descriptively only and deliberately excluded from multivariable modeling because they are non-adjudicated reporting fields that do not establish cause of death or drug attribution. Adjusted estimates for these fields could therefore be misinterpreted as comparative mortality risk. Neither field was interpreted as establishing drug-attributable mortality.

## 3. Results

### 3.1. Cohort and Report Characteristics

The downloaded line listing contained 6368 distinct Case IDs. Of these, 5714 were assigned to SG and 654 to Dato-DXd; no record contained both comparators (Figure 1). The Serious field identified 5648 serious and 720 non-serious reports. Serious reports accounted for 5254 SG reports (91.9%) and 394 Dato-DXd reports (60.2%). Outcome = Died was recorded in 1317 SG reports (23.0%) and 140 Dato-DXd reports (21.4%) (Table 1).

Age was available for 3271 reports (51.4%): 3032 SG reports (53.1%) and 239 Dato-DXd reports (36.5%). Age was unavailable for 3097 reports (48.6%): 2682 SG reports (46.9%) and 415 Dato-DXd reports (63.5%). Among reports with age available, median age was 57 years (IQR, 47–67) for SG and 64 years (IQR, 57–70) for Dato-DXd. Female sex was recorded in 4532 SG reports (79.3%) and 511 Dato-DXd reports (78.1%).

The prespecified indication algorithm classified 3989 reports as breast cancer-related: 3650 SG reports (63.9%) and 339 Dato-DXd reports (51.8%). Healthcare professionals submitted 4974 SG reports (87.0%) and 615 Dato-DXd reports (94.0%). United States event country was recorded for 1573 SG reports (27.5%) and 194 Dato-DXd reports (29.7%). SG report years extended from 2020 through 2026, whereas Dato-DXd reports were concentrated in 2025–2026.

### 3.2. Domain-Level Reporting Profiles

Hematologic cytopenia was reported in 1430 SG records (25.0%) and 27 Dato-DXd records (4.1%). Neutropenia/febrile neutropenia occurred in 1006 SG reports (17.6%) and 15 Dato-DXd reports (2.3%); diarrhea/colitis in 879 (15.4%) and 13 (2.0%), respectively; and infection/sepsis in 620 (10.9%) and 38 (5.8%), respectively (Table 2 and Figure 2).

Conversely, mucositis/stomatitis was reported in 120 Dato-DXd records (18.3%) and 72 SG records (1.3%), and ocular toxicity in 71 (10.9%) and 52 (0.9%), respectively. Exact ILD/pneumonitis occurred in 43 Dato-DXd reports (6.6%) and 126 SG reports (2.2%). Overall gastrointestinal toxicity was similar: 142 Dato-DXd reports (21.7%) and 1283 SG reports (22.5%).

### 3.3. Disproportionality Analysis

For Dato-DXd versus SG, the ROR was 17.61 (95% CI, 12.98–23.90) for mucositis/stomatitis, 13.26 (95% CI, 9.18–19.16) for ocular toxicity, and 3.12 (95% CI, 2.19–4.46) for exact ILD/pneumonitis; each remained significant after FDR correction (Table 2).

Reporting was proportionally lower with Dato-DXd than with SG for hematologic cytopenia (ROR, 0.13; 95% CI, 0.09–0.19), neutropenia/febrile neutropenia (ROR, 0.11; 95% CI, 0.07–0.18), diarrhea/colitis (ROR, 0.11; 95% CI, 0.06–0.19), and infection/sepsis (ROR, 0.51; 95% CI, 0.36–0.71). Overall gastrointestinal toxicity did not differ materially between the groups (ROR, 0.96; 95% CI, 0.79–1.17; FDR q = 0.765).

### 3.4. Adjusted Analyses

After multivariable adjustment in the full-cohort models incorporating a separate missing-age indicator, Dato-DXd remained strongly associated with greater reporting of mucositis/stomatitis (aROR, 20.04; 95% CI, 12.41–32.36) and ocular toxicity (aROR, 13.98; 95% CI, 8.03–24.32) relative to SG (Table 3 and Figure 3).

The aRORs for Dato-DXd versus SG were 0.11 (95% CI, 0.07–0.17) for hematologic cytopenia, 0.12 (95% CI, 0.07–0.20) for neutropenia/febrile neutropenia, 0.11 (95% CI, 0.06–0.19) for diarrhea/colitis, and 0.57 (95% CI, 0.38–0.85) for infection/sepsis. The adjusted reporting association for exact ILD/pneumonitis was attenuated and was no longer statistically significant (aROR, 1.44; 95% CI, 0.87–2.39; FDR q = 0.183). Overall gastrointestinal reporting remained similar (aROR, 1.00; 95% CI, 0.79–1.28; FDR q = 0.973). The common-period sensitivity analysis included 2433 reports received in 2025–2026 (SG, *n* = 1784; Dato-DXd, *n* = 649). The directions and statistical interpretations were consistent with the primary analysis. In particular, exact ILD/pneumonitis remained nonsignificant (aROR, 1.43; 95% CI, 0.80–2.56; FDR q = 0.266), whereas the strong comparative reporting patterns for the remaining toxicity phenotypes were retained (Supplementary Table S3).

### 3.5. Preferred Term-Level Analysis

A total of 1599 distinct PTs were observed; 271 met the ≥10-report threshold and constituted the PT-level FDR family, of which 39 reached FDR significance at q < 0.05 (Supplementary Table S2). Among PTs with at least 10 reports, Stomatitis occurred in 105 Dato-DXd reports (16.1%) and 34 SG reports (0.6%; ROR, 31.95), and Dry Eye in 23 (3.5%) and 6 (0.1%), respectively (ROR, 34.68). Keratitis, Vision Blurred, and Oral Pain also showed proportional reporting enrichment with Dato-DXd (Table 4). Interstitial Lung Disease occurred in 27 Dato-DXd reports (4.1%) and 54 SG reports (0.9%; ROR, 4.51). The exact ILD/pneumonitis phenotype comprised the MedDRA PTs Interstitial Lung Disease and Pneumonitis, with each report counted once at the phenotype level; complete standalone results for both PTs are provided in Supplementary Table S2. The lower ROR for the composite exact ILD/pneumonitis phenotype compared with the Interstitial Lung Disease PT alone (3.12 vs. 4.51) reflects the inclusion of the broader Pneumonitis PT, for which the between-drug reporting contrast was less pronounced.

Table 4 presents a clinically interpretable subset of the PT-level results; complete results for all 271 eligible PTs, including the statistical test used for each PT, are provided in Supplementary Table S2. Fisher’s exact test was used for sparse comparisons, as described in Methods; FDR q values were Benjamini–Hochberg adjusted across all 271 PTs. Operational or administrative PTs were included in the complete PT-level screening presented in Supplementary Table S2 but were excluded from Table 4 because they do not represent clinical toxicity signals. Preferred Term (PT) names are reproduced verbatim from MedDRA; therefore, spelling may differ from the descriptive phenotype labels used elsewhere in the manuscript (e.g., “Diarrhoea” versus “diarrhea/colitis”).

Diarrhoea occurred in 718 SG reports (12.6%) and 6 Dato-DXd reports (0.9%; ROR, 0.06), while Neutropenia occurred in 626 (11.0%) and 9 (1.4%), respectively (ROR, 0.11). Febrile Neutropenia, Neutrophil Count Decreased, Myelosuppression, and Asthenia likewise showed proportional reporting enrichment with SG. Administrative or operational PTs were screened but were not interpreted as clinical toxicity signals.

### 3.6. Breast Cancer Subset

The breast cancer-restricted analysis included 3989 reports (SG, *n* = 3650; Dato-DXd, *n* = 339). Dato-DXd showed higher crude reporting odds for mucositis/stomatitis (21.8% vs. 1.6%; ROR, 17.29; 95% CI, 12.00–24.93) and ocular toxicity (12.1% vs. 1.0%; ROR, 13.43; 95% CI, 8.48–21.28). Exact ILD/pneumonitis was also more frequently reported with Dato-DXd (4.7% vs. 2.0%; ROR, 2.39; 95% CI, 1.38–4.16).

Conversely, Dato-DXd showed lower crude reporting odds for hematologic cytopenia (2.7% vs. 29.0%; ROR, 0.07; 95% CI, 0.03–0.13), neutropenia/febrile neutropenia (1.2% vs. 20.5%; ROR, 0.05; 95% CI, 0.02–0.12), diarrhea/colitis (1.2% vs. 18.5%; ROR, 0.05; 95% CI, 0.02–0.14), and infection/sepsis (5.6% vs. 11.5%; ROR, 0.46; 95% CI, 0.29–0.74). Overall gastrointestinal toxicity was similar between the groups (24.8% vs. 26.6%; ROR, 0.91; 95% CI, 0.70–1.17). Complete *p* values and FDR-adjusted q values are provided in Supplementary Table S4.

### 3.7. Death-Related Reporting

Outcome = Died was recorded in 1317 SG reports (23.0%) and 140 Dato-DXd reports (21.4%). Death as a MedDRA reaction PT was recorded in 762 SG reports (13.3%) and 35 Dato-DXd reports (5.4%). These fields were reported descriptively only and were not subjected to multivariable modeling or interpreted as evidence of drug-attributable mortality.

## 4. Discussion

In this comparative analysis of 6368 distinct Case IDs in the FDA AEMS line listing, SG and Dato-DXd showed clearly differentiated adverse-event reporting profiles. Dato-DXd reports were strongly enriched for mucosal and ocular events, whereas SG reports were enriched for hematologic cytopenias, neutropenia, diarrhea/colitis, and infection/sepsis. Overall gastrointestinal reporting was similar despite substantial differences in the specific gastrointestinal phenotypes represented. Exact ILD/pneumonitis was more frequently reported with Dato-DXd in the crude comparison, but this comparative reporting association was attenuated after adjustment in the full-cohort model incorporating a separate missing-age indicator. The breast cancer-restricted crude ROR analysis was directionally consistent with the full-cohort comparisons (Supplementary Table S4). Together, these findings describe relative reporting enrichment within a two-drug pharmacovigilance cohort rather than comparative incidence or patient-level risk.

The relative predominance of hematologic and diarrheal events with SG is consistent with prospective and postmarketing evidence. In ASCENT-03, neutropenia, diarrhea, and leukopenia were among the most frequent grade 3 or higher adverse events [6]. Previous pharmacovigilance analyses have also identified neutropenia, febrile neutropenia, diarrhea, colitis, and sepsis among the principal SG reporting signals [14,15]. In the present study, these differences were evident at both the phenotype and individual PT levels and persisted after multivariable adjustment. The findings therefore reinforce established surveillance priorities for SG, including blood-count monitoring and early recognition of diarrhea and infectious complications, as reflected in clinical trial experience and prescribing information [16,17]. They should not, however, be translated into estimates of event frequency among treated patients.

Mucosal and ocular events were the most distinctive components of the Dato-DXd reporting profile. Mucositis/stomatitis was reported in 18.3% of Dato-DXd records versus 1.3% of SG records, and ocular toxicity in 10.9% versus 0.9%. Stomatitis, oral pain, dry eye, keratitis, and blurred vision also showed reporting enrichment at the PT level. These observations are aligned with TROPION-Breast02 and with clinical management recommendations that emphasize preventive oral care, monitoring for ocular surface symptoms, and prompt evaluation of clinically relevant events during Dato-DXd treatment [7,13,18]. The present analysis does not establish event grade, duration, or treatment attribution; rather, it shows that these event categories occupy a substantially greater share of Dato-DXd than SG reports.

The gastrointestinal findings illustrate the importance of phenotype granularity. Overall gastrointestinal reporting did not differ materially between the agents, yet the constituent events were distributed very differently: SG reports were dominated by diarrhea/colitis, whereas Dato-DXd reports showed greater representation of oral mucosal events. A broad organ-level category would therefore obscure clinically meaningful differences in presentation and supportive-care requirements. Combining prespecified phenotypes with PT-level screening provided a more clinically interpretable description of these patterns, consistent with the toxicity priorities reported in prospective studies [6,13].

The pulmonary findings warrant greater caution. The crude ROR indicated relative reporting enrichment of exact ILD/pneumonitis with Dato-DXd, and the Interstitial Lung Disease PT likewise showed reporting enrichment. After adjustment for continuous age, a missing-age indicator, and other available report-level characteristics, however, the estimate moved toward the null and was no longer statistically significant. This attenuation may reflect measured differences in indication, calendar time, and reporting context, but residual confounding remains unavoidable. Dato-DXd reports were concentrated in later years and included lung cancer indications, whereas SG had a longer reporting history dominated by breast cancer. This reporting-maturity imbalance may also have been influenced by the Weber effect, whereby spontaneous adverse-event reporting can be disproportionately elevated during the early postmarketing period after a drug’s introduction [19]. Although adjustment for report year accounted for average secular calendar-time variation, it could not fully capture product-specific differences in time since market entry or surveillance maturity. The consistency of the 2025–2026 common-period sensitivity analysis reduces, but does not eliminate, this concern. The adjusted result must not be interpreted as evidence against the established risk of Dato-DXd-associated ILD/pneumonitis, which can be severe or fatal [7,20]. Instead, it demonstrates that cross-product pulmonary comparisons in spontaneous-report databases are sensitive to case mix, covariate availability, and surveillance context [19].

The contrasting profiles are biologically plausible but cannot be assigned a mechanism from these data. SG and Dato-DXd differ in antibody structure, linker properties, payload, drug-to-antibody ratio, pharmacokinetics, and potential systemic payload exposure [4,5,21,22]. TROP2 is also expressed in several normal epithelial tissues, which may be relevant to off-tumor toxicity [23], while SN-38 exposure is associated with marrow and gastrointestinal toxicity [16]. AEMS contains no pharmacokinetic, tissue-distribution, linker-cleavage, or target-expression information. These considerations should therefore be viewed as contextual hypotheses rather than mechanistic conclusions of the present analysis.

The breast cancer-restricted crude comparative analysis addressed one important source of clinical heterogeneity and yielded RORs directionally concordant with the full-cohort comparisons for all eight phenotypes; seven associations remained significant after FDR adjustment, whereas overall gastrointestinal toxicity remained similar between the agents (Supplementary Table S4). Nevertheless, it did not create a conventional head-to-head comparison. The subset remained heterogeneous with respect to biological subtype, treatment line, previous therapy, disease burden, duration of exposure, and supportive-care practices. These factors were not reliably available in the line listing. The subset should therefore be interpreted as a consistency analysis that reduces, but does not eliminate, confounding by indication.

The marked difference in serious-report proportions—91.9% for SG and 60.2% for Dato-DXd—also illustrates why reporting proportions cannot be read as comparative clinical risk. Product age, reporting requirements, indication, manufacturer processes, reporter behavior, publicity, and recognition of known adverse events can influence whether and how a report enters the database [9,19]. Similarly, Outcome = Died and Death as a reaction PT are reporting fields rather than adjudicated causes of death. In patients with advanced malignancy, death may reflect cancer progression, infection, concomitant therapy, comorbidity, or other causes. Accordingly, we deliberately did not fit adjusted models for these fields, because such estimates could be misinterpreted as comparative mortality risk. FDA guidance accordingly emphasizes that disproportionality is a statistical reporting association, not proof of causality or comparative risk [9,11].

The appropriate clinical interpretation is therefore one of surveillance priorities rather than global safety ranking. During SG treatment, the reported pattern is consistent with established attention to blood counts, febrile neutropenia and infection, and early management of diarrhea [17]. For Dato-DXd, established guidance emphasizes proactive oral care, recognition of stomatitis, assessment of ocular symptoms, and prompt evaluation of new or worsening respiratory symptoms [13,18]. The present findings reinforce these label-based priorities but do not identify which individual patients are most likely to experience a given adverse event and should not be used independently to select one ADC over another.

Strengths of this study include use of report-level data, mutually exclusive assignment to the two ADC cohorts, confirmation of distinct Case IDs, evaluation of clinically defined phenotypes alongside individual PTs, FDR correction, multivariable adjustment, and a prespecified indication-restricted analysis. Presenting both crude and adjusted estimates was particularly informative for ILD/pneumonitis, for which the magnitude of the comparative estimate changed after adjustment.

Several limitations should frame interpretation. First, AEMS lacks reliable exposure denominators; reporting proportions, RORs, and aRORs cannot be interpreted as incidence rates, absolute risks, or conventional head-to-head safety estimates. Second, reporting is affected by underreporting, selective and stimulated reporting, coding variability, missing information, notoriety bias, and residual confounding [11,19]. Although the models included a United States event-country indicator, 72.3% of reports had a non-U.S. or unspecified event country; therefore, differences between the two drugs in international reporting practices, regulatory timelines, and market-entry dates may have contributed to the observed reporting patterns beyond what this adjustment could capture. The available export did not distinguish primary from secondary suspect roles, and distinct Case IDs do not exclude duplicate or parallel submissions of the same clinical event. Third, dose, cumulative exposure, treatment duration, line of therapy, baseline comorbidities, laboratory values, event grade, time to onset, management, dechallenge, rechallenge, and adjudicated causality were absent or insufficiently standardized. Age was missing in 48.6% of the cohort and was more frequently missing for Dato-DXd than for SG (63.5% vs. 46.9%). To retain the full cohort, the models used a separate missing-age indicator rather than excluding reports with unavailable age; no imputation was performed. However, this approach cannot recover unobserved ages or eliminate bias from differential or nonrandom missingness; residual age-related confounding and selection bias therefore remain possible. Finally, the nearly ninefold imbalance in report numbers and shorter market maturity of Dato-DXd limit direct comparability. Because SG served as the reference within a two-drug cohort, an ROR above or below 1 indicates only relative enrichment between these agents and not an absolute signal relative to the full AEMS database.

## 5. Conclusions

SG and Dato-DXd showed distinct adverse-event reporting profiles in this comparative AEMS analysis. SG reports were relatively enriched for hematologic toxicity, neutropenia, diarrhea, and infection, whereas Dato-DXd reports were enriched for stomatitis and ocular events. The crude ILD/pneumonitis reporting association with Dato-DXd was attenuated after adjustment, underscoring the influence of indication, calendar time, and reporting context. These findings complement but do not replace randomized trial and label evidence and should be used to support adverse-event vigilance rather than comparative risk estimation or treatment selection. Exposure-linked observational studies and prospective cohorts are needed to quantify absolute risk and identify patient-level predictors.

## Figures and Tables

**Figure 1:**
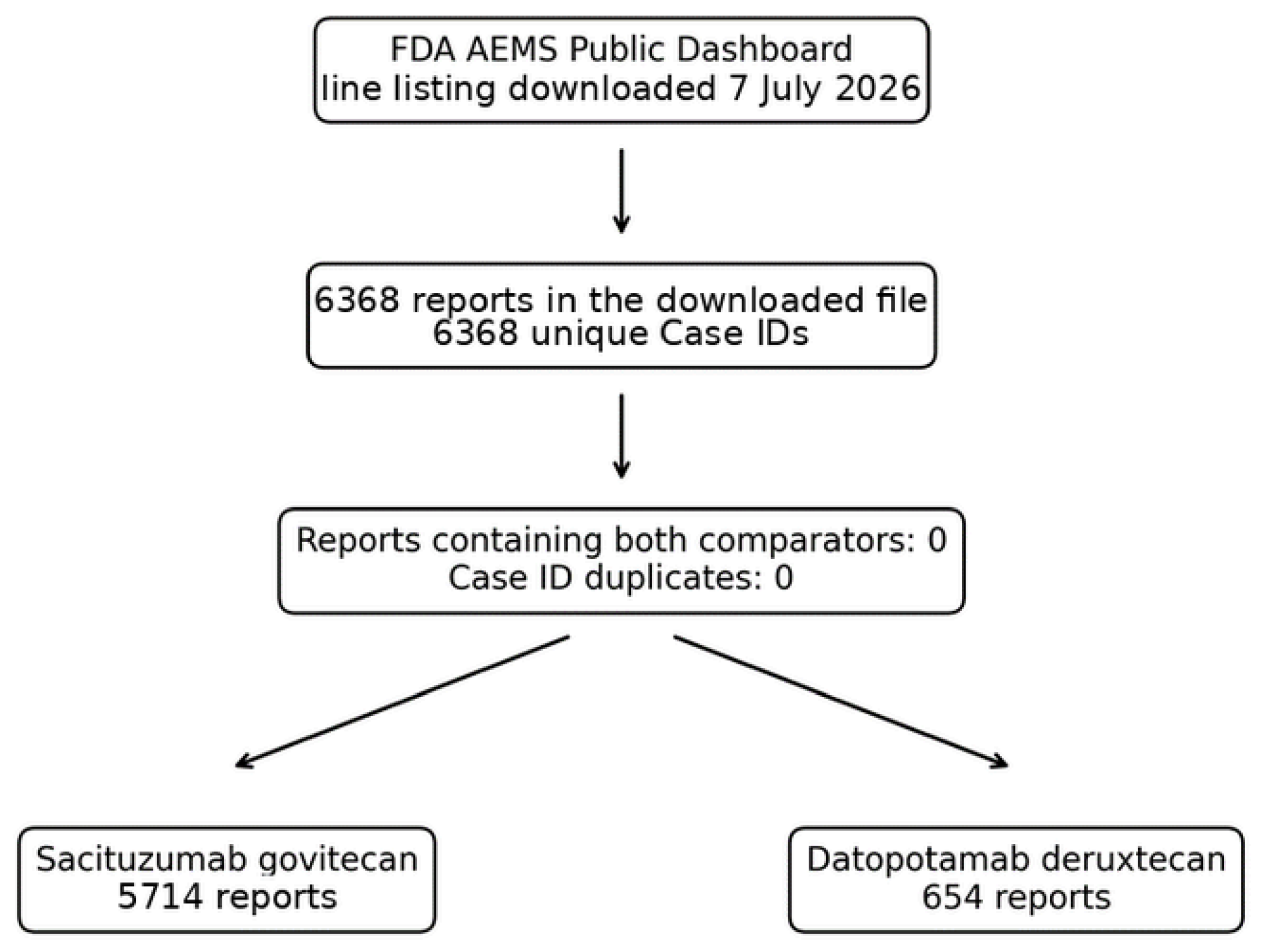
Cohort construction from the FDA AEMS Public Dashboard line listing (Reports were classified according to suspect product name or suspect active ingredient fields. No duplicate Case IDs and no overlapping SG/Dato-DXd reports were found).

**Figure 2:**
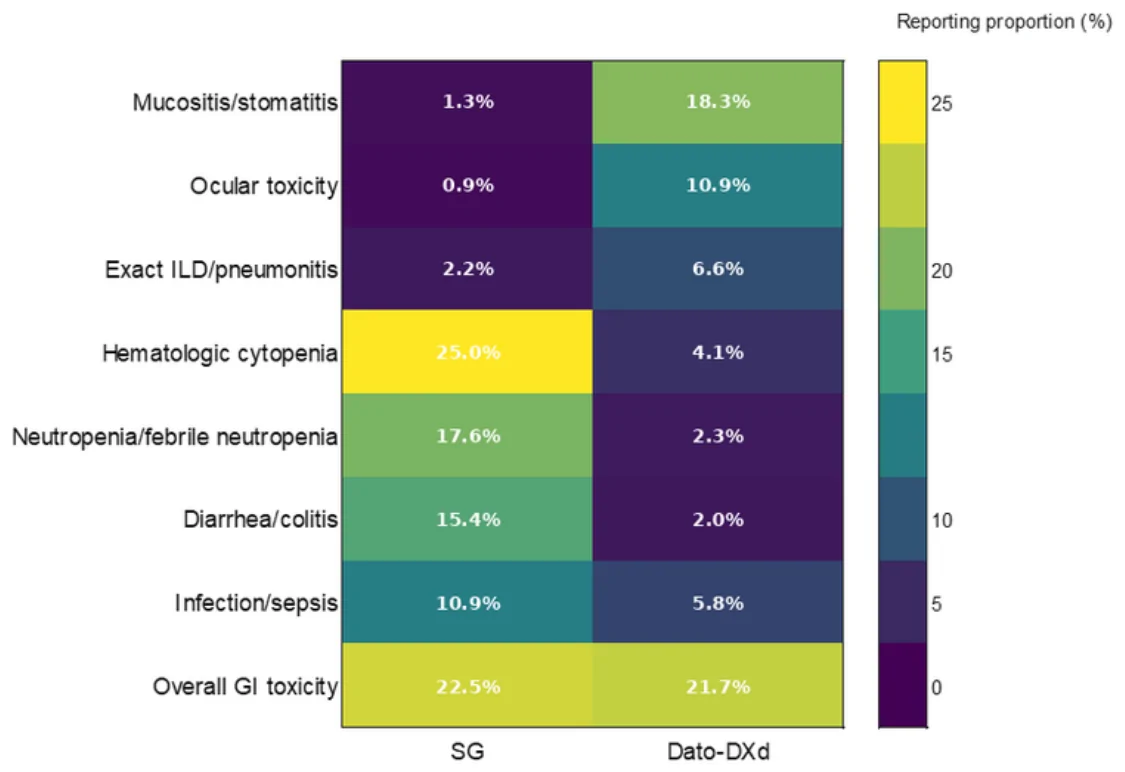
Reporting proportions for selected toxicity phenotypes (The heatmap shows the percentage of reports within each ADC group that contained the corresponding phenotype. These proportions are reporting proportions, not incidence estimates).

**Figure 3:**
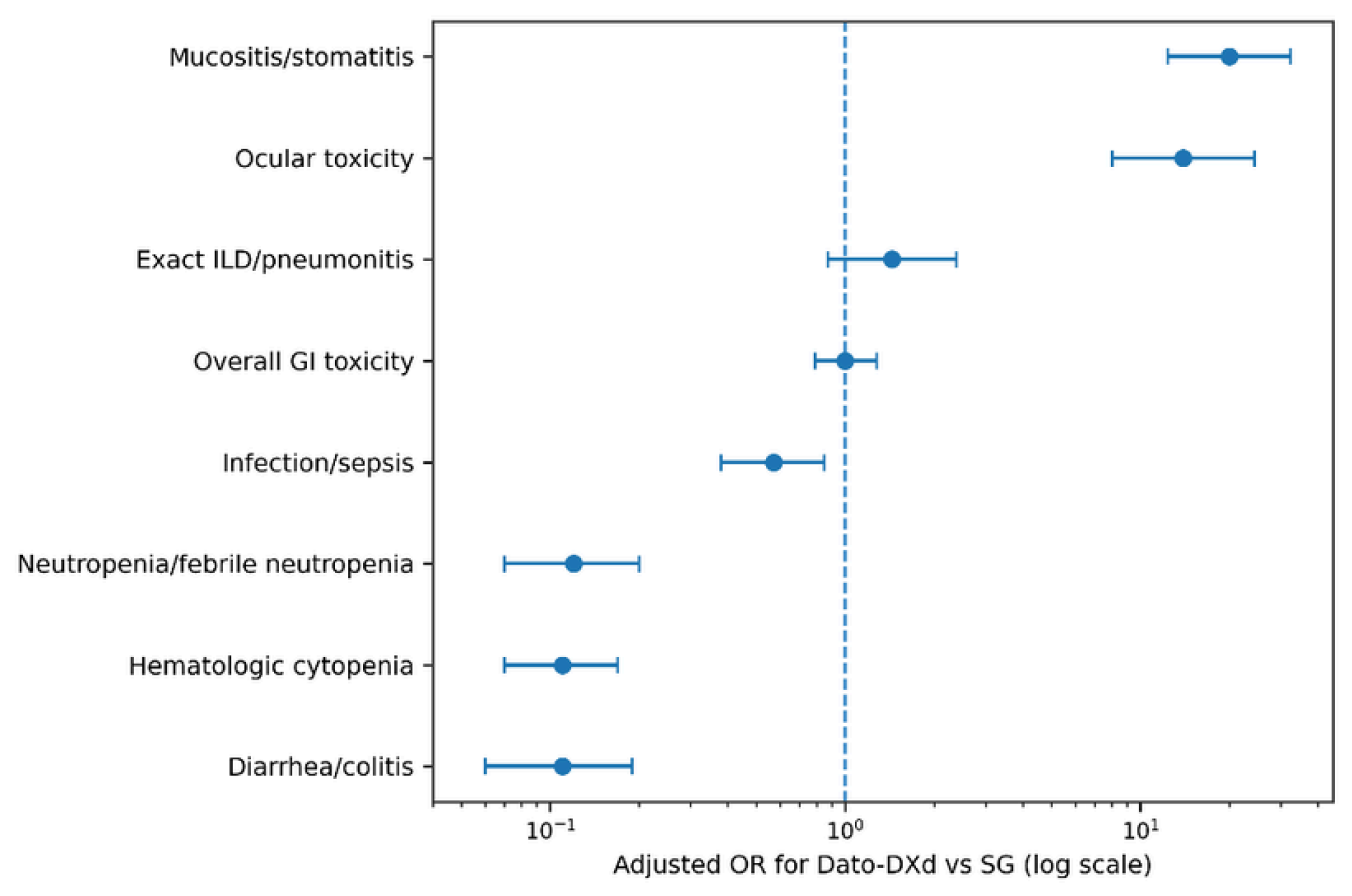
Adjusted reporting odds ratios for selected toxicity phenotypes (Models included the full cohort (N = 6368) with a separate missing-age indicator. Error bars indicate 95% confidence intervals; SG was the reference).

**Table 1:** Cohort and report-level characteristics.

Characteristic	SG (*n* = 5714)	Dato-DXd (*n* = 654)	Total (*n* = 6368)
Reports	5714	654	6368
Serious reports	5254 (91.9)	394 (60.2)	5648 (88.7)
Non-serious reports	460 (8.1)	260 (39.8)	720 (11.3)
Outcome = Died	1317 (23.0)	140 (21.4)	1457 (22.9)
Age available	3032 (53.1)	239 (36.5)	3271 (51.4)
Median age, years (IQR)	57 (47–67)	64 (57–70)	57 (47–67)
Female sex	4532 (79.3)	511 (78.1)	5043 (79.2)
Breast cancer indication	3650 (63.9)	339 (51.8)	3989 (62.6)
Healthcare professional reporter	4974 (87.0)	615 (94.0)	5589 (87.8)
United States event country	1573 (27.5)	194 (29.7)	1767 (27.7)

Abbreviations: Dato-DXd, datopotamab deruxtecan; IQR, interquartile range; SG, sacituzumab govitecan. Outcome = Died and Death PT are reporting variables and were not interpreted as drug-attributable mortality.

**Table 2:** Key toxicity phenotypes: reporting proportions and unadjusted comparative reporting odds ratios.

Toxicity Phenotype	SG *n* (%)	Dato-DXd *n* (%)	ROR	95% CI	FDR q
Mucositis/stomatitis	72 (1.26)	120 (18.35)	17.61	12.98–23.90	<0.01
Ocular toxicity	52 (0.91)	71 (10.86)	13.26	9.18–19.16	<0.01
Exact ILD/pneumonitis	126 (2.21)	43 (6.58)	3.12	2.19–4.46	<0.01
Hematologic cytopenia	1430 (25.03)	27 (4.13)	0.13	0.09–0.19	<0.01
Neutropenia/febrile neutropenia	1006 (17.61)	15 (2.29)	0.11	0.07–0.18	<0.01
Diarrhea/colitis	879 (15.38)	13 (1.99)	0.11	0.06–0.19	<0.01
Infection/sepsis	620 (10.85)	38 (5.81)	0.51	0.36–0.71	<0.01
Overall GI toxicity	1283 (22.45)	142 (21.71)	0.96	0.79–1.17	0.765

ROR values above 1 indicate proportional reporting enrichment with datopotamab deruxtecan; values below 1 indicate proportional reporting enrichment with sacituzumab govitecan. Reporting proportions are not incidence estimates.

**Table 3:** Adjusted comparative reporting odds ratios and underlying report counts for selected toxicity phenotypes.

Phenotype	SG, *n* (%)	Dato-DXd, *n* (%)	aROR	95% CI	*p* Value	FDR q
Mucositis/stomatitis	72 (1.3)	120 (18.3)	20.04	12.41–32.36	<0.001	<0.001
Ocular toxicity	52 (0.9)	71 (10.9)	13.98	8.03–24.32	<0.001	<0.001
Exact ILD/pneumonitis	126 (2.2)	43 (6.6)	1.44	0.87–2.39	0.160	0.183
Hematologic cytopenia	1430 (25.0)	27 (4.1)	0.11	0.07–0.17	<0.001	<0.001
Neutropenia/febrile neutropenia	1006 (17.6)	15 (2.3)	0.12	0.07–0.20	<0.001	<0.001
Diarrhea/colitis	879 (15.4)	13 (2.0)	0.11	0.06–0.19	<0.001	<0.001
Infection/sepsis	620 (10.9)	38 (5.8)	0.57	0.38–0.85	0.006	0.008
Overall GI toxicity	1283 (22.5)	142 (21.7)	1.00	0.79–1.28	0.973	0.973

Counts in the SG and Dato-DXd columns represent phenotype-positive reports contributing to each model, with each report counted once per phenotype; they do not represent event incidence or numbers of individual patients. Percentages were calculated using denominators of 5714 SG reports and 654 Dato-DXd reports. Each of the eight models included the same full cohort (N = 6368; SG, *n* = 5714; Dato-DXd, *n* = 654). Reports with unavailable age were retained using the missing-age-indicator approach described in the Methods. Models were adjusted for age per 10-year increase, a missing-age indicator, sex, report year, breast and lung cancer indications, healthcare professional reporter status, and United States event country. SG was the reference group; q values were Benjamini–Hochberg adjusted across eight models. aROR, adjusted reporting odds ratio; CI, confidence interval; FDR, false discovery rate; ILD, interstitial lung disease.

**Table 4:** Clinically interpretable Preferred Term-level differences among PTs with at least 10 reports.

Adverse Events	Total (*n*)	SG *n* (%)	Dato-DXd *n* (%)	ROR	FDR q
Stomatitis	139	34 (0.60)	105 (16.05)	31.95	<0.01
Dry eye	29	6 (0.11)	23 (3.52)	34.68	<0.01
Keratitis	10	2 (0.04)	8 (1.22)	35.37	<0.01
Vision blurred	26	13 (0.23)	13 (1.99)	8.89	<0.01
Oral pain	14	4 (0.07)	10 (1.53)	22.17	<0.01
Interstitial lung disease	81	54 (0.95)	27 (4.13)	4.51	<0.01
Diarrhoea	724	718 (12.57)	6 (0.92)	0.06	<0.01
Neutropenia	635	626 (10.96)	9 (1.38)	0.11	<0.01
Febrile neutropenia	263	259 (4.53)	4 (0.61)	0.13	<0.01
Neutrophil count decreased	185	183 (3.20)	2 (0.31)	0.09	<0.01
Myelosuppression	137	136 (2.38)	1 (0.15)	0.06	<0.01
Asthenia	302	295 (5.16)	7 (1.07)	0.20	<0.01

## Data Availability

The data analyzed in this study were obtained from the publicly available U.S. Food and Drug Administration Adverse Event Monitoring System (AEMS) Public Dashboard. The processed analytic dataset generated for this study is available from the corresponding author upon reasonable request.

## Supplementary Materials

The following supporting information can be downloaded at: https://www.mdpi.com/article/10.3390/jcm15197405/s1, Table S1: Complete MedDRA Preferred Term definitions for the selected toxicity phenotypes; Table S2: Complete Preferred Term-level comparative analysis for PTs reported in at least 10 records; Table S3: Sensitivity analysis restricted to the common reporting period of 2025–2026; Table S4: Breast cancer-restricted crude comparative reporting analysis for selected toxicity phenotypes.
